# CM from intact hAM: an easily obtained product with relevant implications for translation in regenerative medicine

**DOI:** 10.1186/s13287-021-02607-z

**Published:** 2021-10-12

**Authors:** Antonietta Rosa Silini, Andrea Papait, Anna Cargnoni, Elsa Vertua, Pietro Romele, Patrizia Bonassi Signoroni, Marta Magatti, Silvia De Munari, Alice Masserdotti, Anna Pasotti, Sara Rota Nodari, Giorgio Pagani, Mario Bignardi, Ornella Parolini

**Affiliations:** 1 Centro di Ricerca E. Menni, Fondazione Poliambulanza Istituto Ospedaliero, Brescia, Italy; 2 Department of Life Science and Public Health, Università Cattolica del Sacro Cuore, Rome, Italy; 3 Istituto Zooprofilattico Sperimentale della Lombardia e dell’Emilia Romagna, Brescia, Italy; 4 Department of Gynaecology, Fondazione Poliambulanza, Brescia, Italy; 5 Department of Radiation Oncology, Fondazione Poliambulanza, Brescia, Italy; 6 Fondazione Policlinico Universitario “Agostino Gemelli” IRCCS, Rome, Italy

**Keywords:** Amniotic membrane, Secretome, Conditioned medium, Lyophilization, Immunomodulation, Immune functional assays

## Abstract

**Background:**

It is now well established that factors (free or in extracellular vesicles) secreted by mesenchymal stromal cells (MSC) are important mediators of MSC regenerative actions. Herein we produced the secretome (conditioned medium, CM) from MSC isolated from the amniotic membrane (hAMSC) and CM from the intact amniotic membrane (hAM, no manipulation or enzymatic digestion) in order to potentially identify an effective, easy and less expensive secretome to produce for potential applications in regenerative medicine. Given that immunomodulation is a key mechanism of action through which hAMSC contributes to tissue regeneration, we used a comprehensive panel of in vitro immunomodulatory tests to compare the CMs.

**Methods:**

Amniotic membranes were either cut into fragments or used for hAMSC isolation. CMs from hAMSC at passages 0 and 2 were collected after a standard 5-day culture while CM from hAM was collected after a 2- and 5-day culture. Immunomodulation was assessed in terms of PBMC and T-cell proliferation, T-cell subset polarization, T-regulatory cell induction, cell cytotoxicity and monocyte differentiation toward antigen-presenting cells. Furthermore, we performed a comparison between CM obtained from single donors and pooled CM. We also assessed the impact of lyophilization on the immunomodulatory properties of CM.

**Results:**

We demonstrate that CM from hAM has comparable immunomodulatory properties to CM from hAMSC at passages 0 and 2. Furthermore, we demonstrate that pooled CMs have similar effects when compared to CM from single donors used separately. Finally, we demonstrate that lyophilization does not alter the in vitro immunomodulatory properties of CM from hAM and hAMSC.

**Conclusions:**

The results presented herein support the possibility to produce secretome from intact hAM and open the prospect to highly improve the scalability of the GMP production process while reducing the costs and time related to the process of cell isolation and expansion. Moreover, the possibility of having a lyophilized secretome that maintains its original properties would allow for a ready-to-use product with easier handling, shipping and storage. The use of a lyophilized product will also facilitate clinicians by permitting customized reconstitution volumes and methods according to the most suitable formula required by the clinical application.

## Introduction

The human amniotic membrane (hAM) is a fetal annex that encloses the fetus and contains the amniotic fluid. The hAM is a tissue that mostly comprises mesenchymal stromal cells (hAMSC), epithelial cells (hAEC) and extracellular matrix. hAM under different formulations (intact or decellularized, cryopreserved, dehydrated, lyophilized or micronized) is a therapeutic biomaterial that has been used for decades and is currently used in the clinic, mainly in ophthalmology and dermatology [1].

Over the last two decades, cells obtained from the hAM, and more specifically hAMSC [2], have attracted much attention due to their immunomodulatory properties [3], which offer significant advantages for their use in the treatment of inflammatory, immune-mediated diseases and in the field of regenerative medicine [4].

We and others have significantly contributed to demonstrating that the hAMSC secretome, also referred to as conditioned medium (CM-hAMSC), exerts effects that are comparable to its cellular counterpart. More specifically, bioactive factors released from hAMSC suppress the in vitro proliferation, inflammatory cytokine production, and functions of T lymphocytes [5, 6], monocytes [7], dendritic cells [8] and macrophages [7] and are able to induce a phenotype and functional switch of monocytes toward macrophages with anti-inflammatory, pro-regenerative M2-like features [6, 7] and also support the expansion of regulatory T cells [5, 6]. More recently, we demonstrated that in vitro, CM-hAMSC suppresses B-cell proliferation, and B-cell differentiation, with an increase in mature B cells and a reduction in antibody-secreting cells [9].

CM-hAMSC has been successfully used to treat different preclinical disease models with exacerbated/deregulated inflammation, including lung [10–14] and liver [15] fibrosis, wound healing [7, 16, 17], collagen-induced arthritis [18, 19], multiple sclerosis [18], inflammatory bowel disease [18], colitis [18, 20], sepsis [18], traumatic brain injury [21] and Huntington's disease [22].

Furthermore, when compared to a cellular product, CM could be more easily preserved as an off-the-shelf product.

The aim of our study was to identify a CM obtained from freshly isolated or from cell culture expanded hAMSC and from intact hAM, which could be a candidate for clinical translation in regenerative medicine in terms of effectiveness, simple production process, recovery in large quantities and at a low cost. We also sought to identify a CM that could be “off the shelf”; thus, it is easy to store, ship and use. Since in the field of regenerative medicine immunomodulation is an essential feature of MSC and their CM [23], we used well-established, in vitro immunomodulatory tests to compare different types of CM. Herein, we also performed a comparison between CM obtained from single donors and from pooled CM in order to assess the impact of donor variability and to test the feasibility of obtaining larger volumes of CM while maintaining functional properties. Finally, in order to obtain a concentrated, effective and easily stored product that could be relevant for future clinical translation, we tested if lyophilization could alter the in vitro immunomodulatory properties of CM from hAMSC and hAM.

## Materials and methods

### Ethics statements

The collection of human peripheral blood mononuclear cells (PBMC) for research purposes was approved by the Regional Departments of Transfusion Medicine (Rif. 523, July 7, 2016). PBMC were obtained from healthy adult donors after informed consent and provided by Center of Immune Transfusion of Spedali Civili of Brescia, Italy.

Human term placentae were collected from healthy women after vaginal delivery or caesarean section at term, after obtaining informed written consent, according to the guidelines set by the local ethical committee “Comitato Etico Provinciale di Brescia,” Italy (number NP 2243, January 19, 2016). The number of CMs used for each experiment is indicated in the figure legends.

### hAM fragment preparation

For each placenta, the amniotic membrane was manually separated from the chorion and cut into 100 × 1 cm^2^ fragments. The fragments were decontaminated by placing them in PBS + 0.25% povidone-iodine for 1–2 s, then removed and incubated in PBS + P/S + amphotericin B + cefamezin for 3 min. The fragments were then washed in saline solution containing 100 U/mL penicillin and 100 μg/mL streptomycin (herein referred to P/S, all from Merck, St. Louis, MO, USA) and used as follows:

1. 50 fragments were placed in 50-mL conical tubes with filter cap (Greiner One) in 10 mL of Ultraculture medium (Lonza) and left for 2 days under gentle rotation at 37 °C and 5% CO_2_
2. 50 fragments were placed in 50-mL conical tubes with filter cap (Greiner One) in 10 mL of Ultraculture medium (Lonza) and left for 5 days under gentle rotation at 37 °C and 5% CO_2_

The leftover membrane was cut into 9cm^2^ fragments and used for hAMSC isolation (see below).

### Isolation of mesenchymal stromal cells from human amniotic membrane (hAM)

Cells were isolated as previously described [24]. hAM fragments were digested at 37 °C for 9 min with 2.5 U/mL dispase (VWR, Radnor, PA, USA) and then transferred to RPMI complete medium composed of RPMI 1640 medium supplemented with 10% heat-inactivated fetal bovine serum (FBS), 1% P/S and 1% L-glutamine (all from Sigma-Aldrich, St. Louis, MO, USA). Afterward, the fragments were treated with 0.94 mg/mL collagenase and DNase I (both from Roche, Basel, Switzerland) for approximately 2.5–3 h at 37 °C. The resulting cell suspensions were centrifuged at low g, and the supernatant was filtered through a 100-μm cell strainer (BD Falcon, Bedford, MA, USA), and the cells were collected by centrifugation.

Freshly isolated cells are referred to as hAMSC p0 and were expanded until passage 2 (p2) by plating at a density of 10^4^ cells/cm^2^ in Chang medium C (Irvine Scientific, Santa Ana, CA, USA) supplemented with 2 mM L-glutamine at 37 °C in the incubator at 5% CO_2_.

hAMSC after isolation (p0) and after two culture passages (p2) were phenotypically characterized as previously reported [24]. Cells that had > 80% expression of mesenchymal markers CD13 and CD90, < 10% of hematopoietic marker CD45 and < 10% of epithelial marker CD324 were used in this study.

### Preparation of conditioned medium (CM)

*From hAM fragments:* fragments from intact hAM were left in culture medium for 2 and 5 days under gentle rotation at 37 °C and 5% CO_2_. A total of 17 placentae were used to produce CM from hAM. At the end of incubation, CM was collected, centrifuged at 300×*g*, filtered through a 0.2-μm sterile filter (Sartorius Stedim, Florence, Italy) and kept frozen at − 80 °C until use.

*From hAMSC p0 and p2*: hAMSC was cultured for 5 days in 24-well plates (Corning, NY, USA) at a density of 5 × 10^5^ cells/well in 0.5 mL of Ultraculture complete medium, composed of Ultraculture medium (Lonza), supplemented with 2 mM L-glutamine (Sigma-Aldrich) and 1% P/S (both from Sigma-Aldrich) as described [25]. At the end of incubation, CM was collected, centrifuged at 300×*g*, filtered through a 0.2-μm sterile filter (Sartorius Stedim, Florence, Italy) and kept frozen at − 80 °C until use. A total of 27 and 11 placentae were used to produce CM from hAMSC p0 and p2, respectively.

Each experiment was performed either using CM from single donors or by pooling together CM obtained from at least 3 different hAMSC/hAM donors.

### CM lyophilization and reconstitution

The lyophilization procedure is an entirely automated process. After filling 2.5-mL vials with CM, they were positioned inside the lyophilization chamber (Lyophilizer Pilot MAX MX 8556, Millrock Technology, USA) and subject to an initial freezing step at − 40 °C for 4 h, followed by an additional freezing cycle at − 45 °C under vacuum. Subsequently, a primary dry cycle divided in 7 phases at increasing temperatures, ranging from 10 to 30 °C, under vacuum, was performed for 13 h. The procedure was completed with a second dry cycle under vacuum at 30 °C for one hour. The entire lyophilization protocol lasted 19 h and was considered complete only when the product had reached a temperature of 25 °C for at least 1 h. Immediately before use, the lyophilized CM was reconstituted with 2.5 mL of sterile water and filtered through a 0.2-μm sterile filter (Sartorius Stedim, Florence, Italy).

### Analysis of T cell proliferation

T cell proliferation was induced by stimulating PBMC through T cell receptor by the addition of anti-CD3 or in mixed lymphocyte culture. Human PBMC were obtained from heparinized whole blood samples using density gradient centrifugation (Histopaque 1077, Sigma-Aldrich, St. Louis, MO, USA).

PBMC (1 × 10^5^/well in a 96-well-plate) were activated with 125 ng/mL (final concentration) anti-CD3 (Orthoclone OKT3, Janssen-Cilag, Cologno Monzese, Italy). Activated PBMC (PBMC + anti-CD3) were cultured in the presence of 50 or 100 μL/well of CM (25% or 50%, respectively, of the final volume), for 3 days with the exception of the results presented in Fig. 4A, where also the 10 μL/well (5%) concentration was used. The final volume of each well was 200 μL. In all experiments, activated PBMC cultured alone were used as controls. All conditions were performed in triplicate in RPMI 1640 medium (Cambrex, Verviers, Belgium) supplemented with 10% heat-inactivated FBS, 2 mM L-glutamine and P/S.

T cell proliferation was assessed by 5-ethynyl-2′-deoxyuridine (EdU) incorporation as previously described [6]. Briefly, 10 µM EdU (Life Technologies, Carlsbad, CA, USA) was added to PBMC at day 3 post-stimulation. After 16–18 h, cells were harvested and EdU incorporation was evaluated by adding 2.5 μM 3-azido-7-hydroxycoumarin (Jena Biosciences, Jena, Germany) in buffer solution (100 mM Tris–HCl pH 8.0, 10 mM L-ascorbic acid, 2 mM CuSO_4_) at RT for 30 min. Cells were acquired using a FACSAria III (BD Biosciences), and the percentage of proliferating EdU-positive cells was analyzed with FCS express v5 (De Novo Software, Los Angeles, CA, USA). Cells were also stained with E-Fluor 780 (Thermofisher) for the exclusion of dead cells.

Alternatively, T cell proliferation was induced in mixed lymphocyte reaction (MLR-T) by co-culture of T cells (1 × 10^5^), isolated from PBMC using the Pan Isolation Kit (Miltenyi), with 1 × 10^5^ gamma-irradiated allogeneic PBMC. Co-culture was performed in the absence or presence of CM (100, 50, or 10 μL/well; 50%, 25% or 5%, respectively, of the final volume). T cell proliferation was evaluated after 6 days of co-culture by CFSE CellTracer™ CFSE Cell Proliferation Kit (Invitrogen) following manufacturer’s instructions. T cell proliferation was analyzed by flow cytometry and is expressed as percent of CFSE diluting cells (proliferative fraction PF: representative of proliferating cells).

### Phenotype of CD4^+^ T helper (Th) and T regulatory (Treg) subsets

Phenotypes were assessed by flow cytometry analysis of the expression of specific cell surface markers and transcription factors to identify T helper subsets (Th1, Th2 and Th17) and Treg. After 5 days of co-culture in the presence of CM, PBMC stimulated with anti-CD3 or T cells stimulated with allogeneic PBMC (MLR-T) were collected and centrifuged at 300*g* for 5 min. Cells were stained with E-Fluor 780 (Thermofisher) for the exclusion of dead cells. The staining was performed using antibodies for CD3 (clone UCHT1), CD4 (clone VIT-4), CD45RA (clone HI100), CD196 (clone 11A9), CD183 (clone 1C6/CXCR3), CD25 (clone M-A25), which all came from BD Biosciences and CD194 (clone REA279) from Miltenyi. Intracellular staining for the transcription factor FoxP3 was performed after fixation and permeabilization using BD Cytofix/Cytoperm (BD Biosciences), followed by staining with anti-FoxP3 antibody (clone R16-715, BD Biosciences). Samples were acquired using a FACSAria III (BD Biosciences) and analyzed with FCS express v5 (De Novo Software, Los Angeles, CA, USA). T cell subsets were identified by a sequential gating strategy. T effector cells were first identified by gating CD4 positive and CD45RA negative cell populations (CD4^+^CD45RA^−^ cells) and different Th subsets were identified as follows: Th1 as CD196^−^CD183^+^, Th17/Th1 as CD196^+^CD183^+^, Th2 as CD196^−^CD183^−^CD194^+^ and Treg as CD25^hi^FoxP3^+^ [26].

### Cytotoxicity marker expression

To analyze the ability of CM to modulate the expression of cytotoxicity markers on PBMC activated with anti-CD3, 1 × 10^5^ PBMC were cultured in presence of CM (100, 50 μL/well; 50% or 25% of the final volume).

For degranulation and cytotoxicity, PBMC (1 × 10^5^), activated with anti-CD3 for 3 days, and T cells (1 × 10^5^) activated with PBMC (MLR-T) for 5 days, in the presence or absence of CM, were stimulated with PMA/ionomicin in the presence of CD107a (clone H4A3) and GolgiStop (BD Biosciences). After 1 h and 15 min, brefeldin A was added.

After an additional 5.5 h, cells were stained with E-Fluor 780 (Thermofisher) for the exclusion of dead cells and with CD3 (clone UCHT1), CD8 (clone SK1), CD14 (clone MφP9, all from BD Biosciences) and CD56 (clone N901, BeckmanCoulter). CD4^+^ T lymphocytes are represented by CD3^+^CD8^−^ cells. The intracellular staining for perforin (clone δG9), granzyme B (GrzB) (clone GB11) and IFN-γ (clone B27) was performed upon fixation and permeabilization with BD cytofix/cytoperm (all from BD Biosciences). Cells were acquired using a FACSAria III (BD Biosciences) and analyzed with FCS express v5 (De Novo Software, Los Angeles, CA, USA).

### Analysis of monocyte differentiation toward antigen-presenting cells

For dendritic cell (DC) differentiation, 2.5 × 10^5^ PBMC were cultured in 48-well plates for four days (Corning) in the presence of 50 ng/mL recombinant human IL-4 (R&D Systems, Minneapolis, MN, USA) and 50 ng/mL granulocyte macrophage colony stimulating (GM-CSF, Miltenyi Biotec) in 0.5 mL RPMI 1640 complete medium (Sigma Aldrich). Complete maturation was reached by adding 0.1 μg/mL lipopolysaccharide (LPS, Sigma Aldrich) for two days.

Monocyte-derived M1 macrophages were obtained from 5 × 10^5^ PBMC cultured in 24-well plates for four days (Corning) in the presence of 5 ng/mL GM-CSF (Miltenyi Biotec) in 0.5 mL RPMI 1640 complete medium (Sigma Aldrich). Cells were activated by adding 20 ng/ml interferon gamma (IFN-γ) for 1 h after which 0.1 μg/mL LPS) (Sigma-Aldrich) was added and left for two days.

Mature dendritic cells (mDC) and M1 macrophages were collected after 6 days of differentiation in the absence or presence of 50 or 100 μL/well of CM (10% or 20%, respectively, of the final volume). Different CM products were added at day zero, concomitantly with the start of the differentiation protocol. The phenotypic profile was investigated by flow cytometry. Prior to surface marker staining, cells were stained with E-Fluor 780 (Thermofisher) for the exclusion of dead cells. Cells stained with CD3 were excluded; CD11b (clone ICRF44) positive cells were analyzed for CD206 (clone 19.2), CD163 (clone GHI/61), CD209 (clone DCN46), CD197 (clone 3D12), CD83 (clone HB15e) and CD14 (clone MΦP9) expression (all antibodies purchased from BD Biosciences).

### Statistical analysis

The data are shown as violin truncated plots with Tukey variations. The parameters were compared using one-way and two-way analysis of variance (ANOVA). Data are representative of at least three independent experiments. Statistical analysis was performed using Prism 8 (GraphPad Software, La Jolla, CA, USA). A *p* value lower than 0.05 was considered statistically significant.

## Results

### Comparison of the ability of conditioned media from hAMSC and hAM to inhibit the proliferation of activated PBMC

**Fig. 1:**
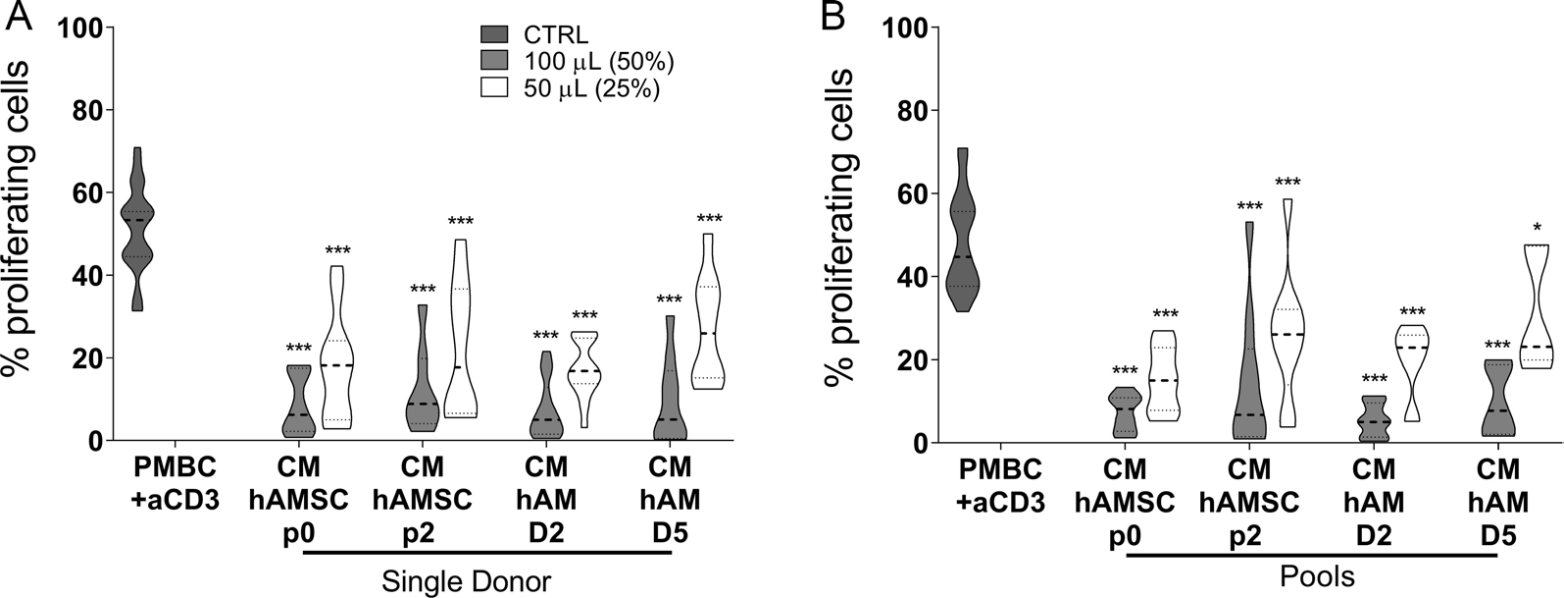
Comparison of conditioned media from single and pooled donors of amniotic membrane derivatives on PBMC proliferation. Allogeneic PBMC (1 × 10^5^) were stimulated with anti-CD3 antibody in the presence of 100 µL (50%) or 50 µL (25%) of conditioned medium (CM) from hAMSC at passage 0 (CM-hAMSC p0) or passage 2 (CM-hAMSC p2), or CM from hAM collected 2 or 5 days after culture (CM-hAM D2 and CM hAM D5, respectively). Results are expressed as percentage of cell proliferation. PBMC stimulated with anti-CD3 mAb constitute the positive control. CMs were either kept separately (**A**, single donors) or pooled (**B**) from multiple donors. Results are displayed as violin plots showing median (thick line), 25th and 75th quartiles (*p < 0.01, ***p < 0.001 versus control (PBMC + anti-CD3), **A**: *N* ≥ 7; **B**: *N* ≥ 4 individual experiments

We compared CM from hAMSC and from hAM fragments, obtained from either single (Fig. 1A) or pooled donors (Fig. 1B), for their ability to inhibit the proliferation of PBMC activated with anti-CD3. First, we observed that CM from hAMSC at passage 0 and passage 2 was able to significantly inhibit proliferation at both doses used (50 and 100 µL, 25% or 50%, respectively, of the final volume) (Fig. 1A). Furthermore, CM from hAM collected after 2 and 5 days had comparable effects to the CM obtained from hAMSC. Interestingly, when CM was pooled together from different hAMSC or hAM, the significant inhibition of PBMC proliferation was maintained (Fig. 1B).

### Comparison of the ability of conditioned media from hAMSC and hAM to modulate T-cell subsets

**Fig. 2:**
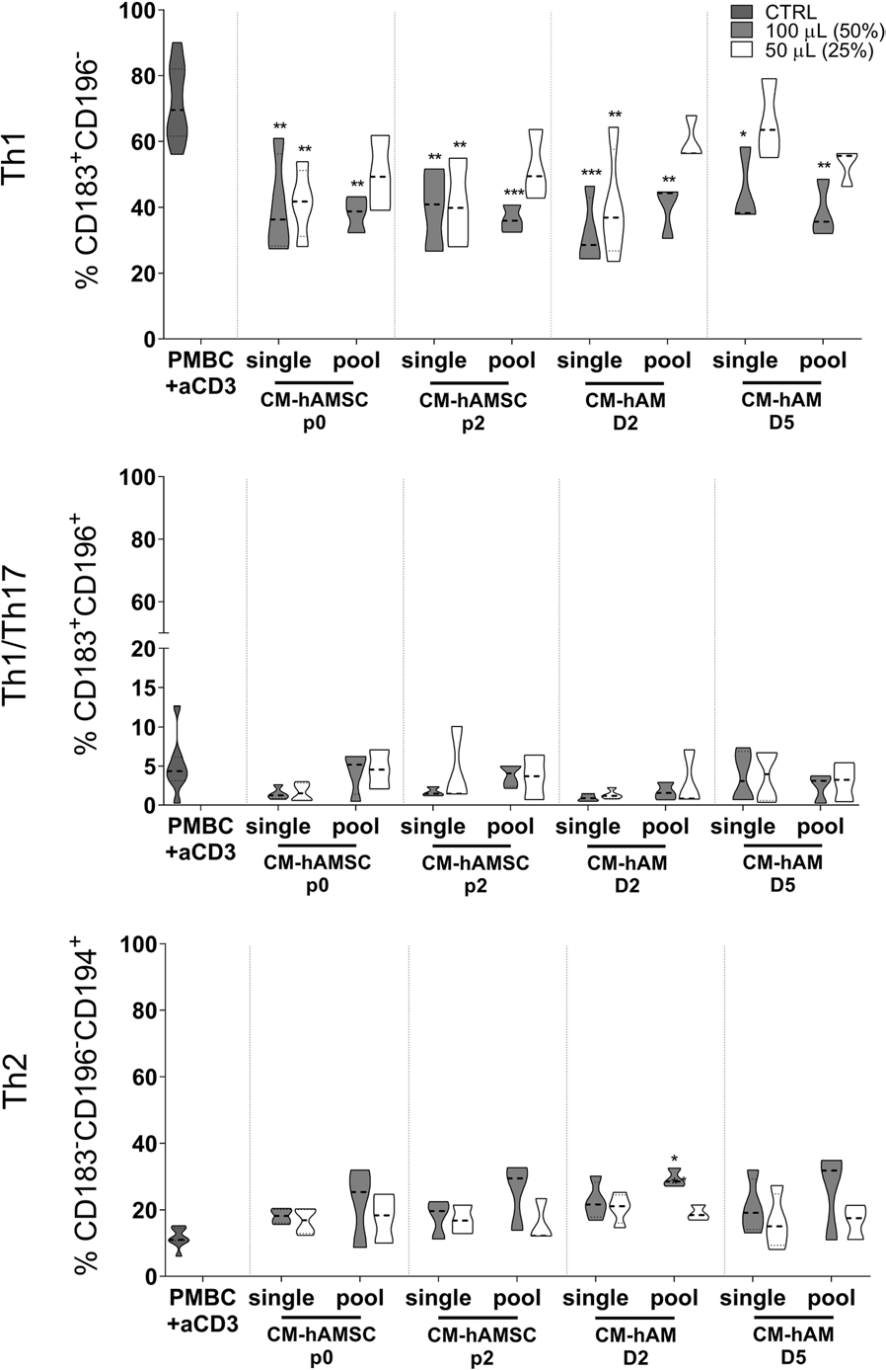
Effect of conditioned media from single and pooled donors of from amniotic membrane derivatives on Th1/Th2/Th17 polarization. Allogeneic PBMC were stimulated with anti-CD3 mAb and co-cultured with 100 µL (50%) or 50 µL (25%) of conditioned medium (CM) from hAMSC at passage 0 (CM-hAMSC p0) or passage 2 (CM-hAMSC p2), or CM from hAM collected 2 or 5 days after culture (CM-hAM D2 and CM hAM D5, respectively). CMs were either kept separately or pooled from multiple donors. Th1 (CD183+CD196−), Th1/Th17 (CD183+CD196+) and Th2 (CD183-CD196-CD194+) phenotype was evaluated. Results are displayed as violin plots showing median (thick line), 25th and 75th quartiles (*p < 0.05, **p < 0.01, ****p < 0.0001), *N* ≥ 3 individual experiments

We then sought to analyze whether CM from hAMSC and from hAM fragments obtained from single and pooled donors were able to modulate T-cell subsets (Fig. 2). We observed that CM from single donors of hAMSC (CM-hAMSC p0 and CM-hAMSC p2) and CM from single donors of hAM (collected after a 2-day culture) were all able to significantly decrease Th1 cells at both doses used (50% and 25%). Concomitantly with the reduced switch to Th1 cell phenotype, we also observed that CM addition markedly reduced the levels of Th1-related cytokines (IFN-γ and TNF-α) in the supernatants of activated PBMC (data not shown).

CM from single donors of hAM collected after a 5-day culture had an effect only at the highest dose used (50%) (Fig. 2, upper panel). Concerning the effects of CM from pooled donors, the reduction in Th1 cells was weaker when compared to CM from single donors (with the exception of CM-hAM D5), but yet still significant (at highest dose used, 100 µL, corresponding to 50% of the final volume), when compared to PBMC stimulated with anti-CD3 (Fig. 2A, upper panel).

Furthermore, we did not observe any significant differences on the Th1/Th17 subsets (Fig. 2, middle panel). On the contrary, all CMs from both single and pooled donors were able to slightly increase the percentage of Th2 cells; this effect was significant only when CM from pooled donors of hAM, collected after a 2-day culture, was used (Fig. 2, lower panel). Altogether, these results suggest that CM from hAMSC and hAM, both from single donors and from pools, had comparable effects on T-cell subsets.

### Comparison of the ability of conditioned media from hAMSC and hAM to modulate T- and NK-cell cytotoxicity

In order to further compare CM from single donors and from pools, we then sought to analyze their effect on cell cytotoxicity.

**Fig. 3:**
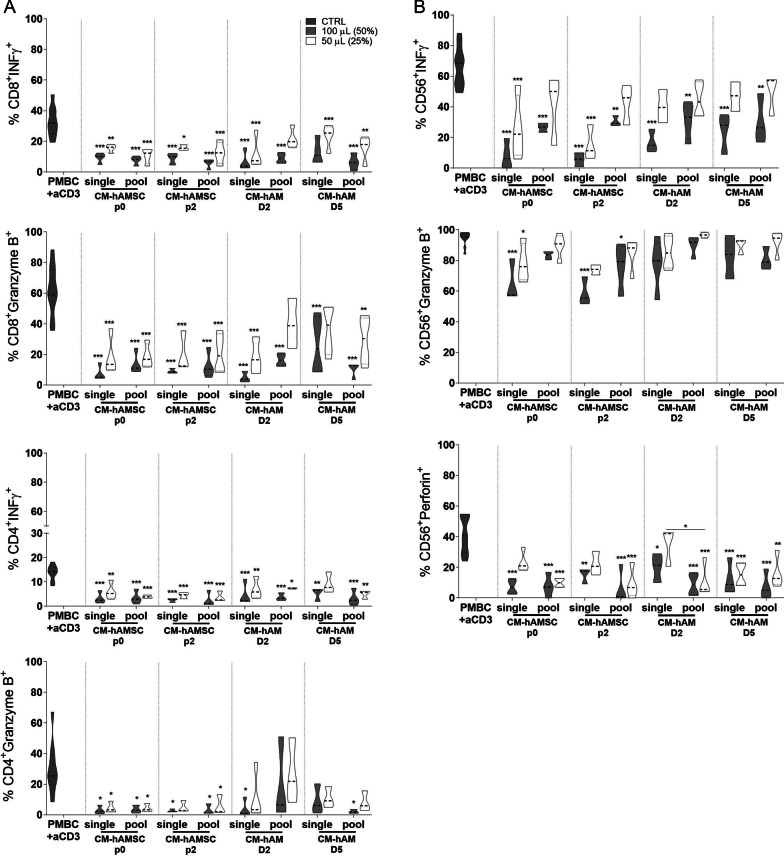
Effect of conditioned media from single and pooled donors of amniotic membrane derivatives on cytotoxicity marker expression by (**A**) T lymphocytes and (**B**) NK cells. Allogeneic PBMC were incubated with anti-CD3 mAb in the presence of 100 µL (50%) or 50 µL (25%) of conditioned medium (CM) from hAMSC at passage 0 (CM-hAMSC p0) or passage 2 (CM-hAMSC p2), or CM from hAM collected 2 or 5 days after culture (CM-hAM D2 and CM hAM D5, respectively). CMs were either kept separately or pooled from multiple donors. The percentage of IFN-γ, granzyme B and perforin positive cells within the (**A**) CD4+, CD8+ T cell and (**B**) CD3-CD56+ NK cell populations was assessed by flow cytometry. Results are displayed as violin plots showing median (thick line), 25th and 75th quartiles (*p < 0.05, **p < 0.01, ***p < 0.001, versus control (PBMC + CD3), *N* ≥ 3 individual experiments

We first analyzed CD8 and CD4 T-cell cytotoxicity in terms of IFN-γ and granzyme B expression (Fig. 3A). We observed that CM from hAMSC and hAM, single donors and pools, was all able to significantly decrease IFN-γ and granzyme B expression on CD8 T cells, and the effects were comparable between single donors and pools. In addition, this was also observed for CD4 T-cell cytotoxicity whereby all CMs analyzed significantly reduced the expression of the common cytotoxicity markers (Fig. 3A).

We then analyzed NK-cell cytotoxicity in terms of IFN-γ, granzyme B and perforin expression (Fig. 3B). CM from hAMSC and hAM were all able to significantly decrease IFN-γ and perforin expression on NK cells, and the effects were comparable between single donors and pools. The effects on granzyme were less evident, whereas a slight and non-significant decrease was observed, with the exception of CM from single donors of hAMSC at p0 and p2, both of which significantly reduced granzyme expression on NK cells.

Given that, for the majority of the parameters analyzed, CM from single donors and that from pools had comparable immunomodulatory capacity, we carried out subsequent studies with pools only.

### Lyophilization does not alter the immunomodulatory properties of conditioned media from hAMSC and hAM

In order to explore the impact of lyophilization on CM immunomodulatory activity, we compared non-lyophilized CMs (Cryo: cryopreserved conditioned medium) and lyophilized (Lyo) CMs. In this experimental setup, we excluded CM-hAM collected at day (D2) in order to examine CMs obtained from the same day (5 days) of culture.

**Fig. 4:**
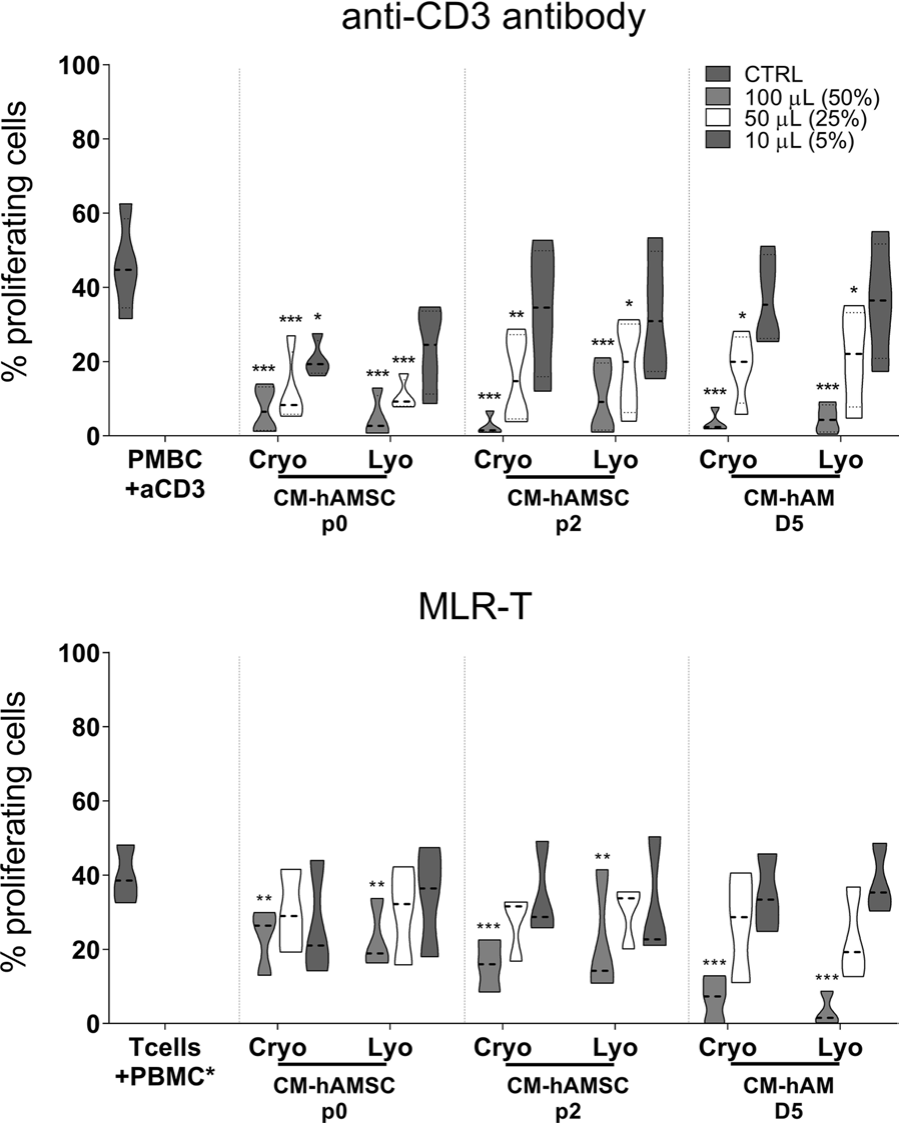
Comparison of lyophilized and cryopreserved CM on T-cell proliferation, Allogeneic PBMC were stimulated with anti-CD3 mAb (upper panel) or in mixed T lymphocyte reaction (MLR-T, bottom panel) in the presence of three different concentrations of cryopreserved or lyophilized CM, 100 µL (50%), 50 µL (25%) or 10 µL (5%), from hAMSC at passage 0 (CM-hAMSC p0) or passage 2 (CM-hAMSC p2), or CM from hAM collected at 5 days after culture (CM hAM D5). Results are represented as violin plots showing median (thick line), 25th and 75th quartiles (*p < 0.05, **p < 0.01, ****p < 0.0001 versus control PBMC + antiCD3 or T cells + PBMC*), *N* ≥ 3 individual experiments

First, we compared the capacity of Lyo-CMs and Cryo-CMs to inhibit T cell proliferation induced either by PBMC stimulation with anti-CD3 (Fig. 4, top panel) or in MLR-T by co-culture with irradiated allogeneic PBMC, the latter of which represents a more physiological setting (Fig. 4, bottom panel).

We found that lyophilized CMs maintained a similar capacity to inhibit T cell proliferation when compared to their non-lyophilized counterparts, both in PBMC stimulated with anti-CD3 and in the MLR-T setting. PBMC viability was not affected by the addition of all CMs. As a matter of fact, the viability of PBMC was 80.3 ± 7.5% and 83.4 ± 6.4% (in the presence or absence of the proliferative stimulus αCD3, respectively), and the addition of cryopreserved or lyophilized CMs (100 µL = 50% of test volume) from hAMSC/P0 (79.9 ± 9.7% or 83.5 ± 6.1%), from hAMSC/P2 (78.8 ± 8.8% or 80.8 ± 9.0%) and from intact hAM (78.3 ± 10.5% or 78.0 ± 8.2%) did not significantly change this parameter (data not shown).

Interestingly, in anti-CD3 stimulated PBMC, all CMs reduced T cell proliferation when used at both doses (100 µL and 50 µL; 50% and 25% of the final volume, respectively) (Fig. 4, top panel), while in the MLR-T setting, only the higher doses of lyophilized or cryopreserved CMs were able to significantly reduce T cell proliferation (Fig. 4, bottom panel), suggesting that CMs inhibitory activity could be related to the stimulus used to induce T cell proliferation.

**Fig. 5:**
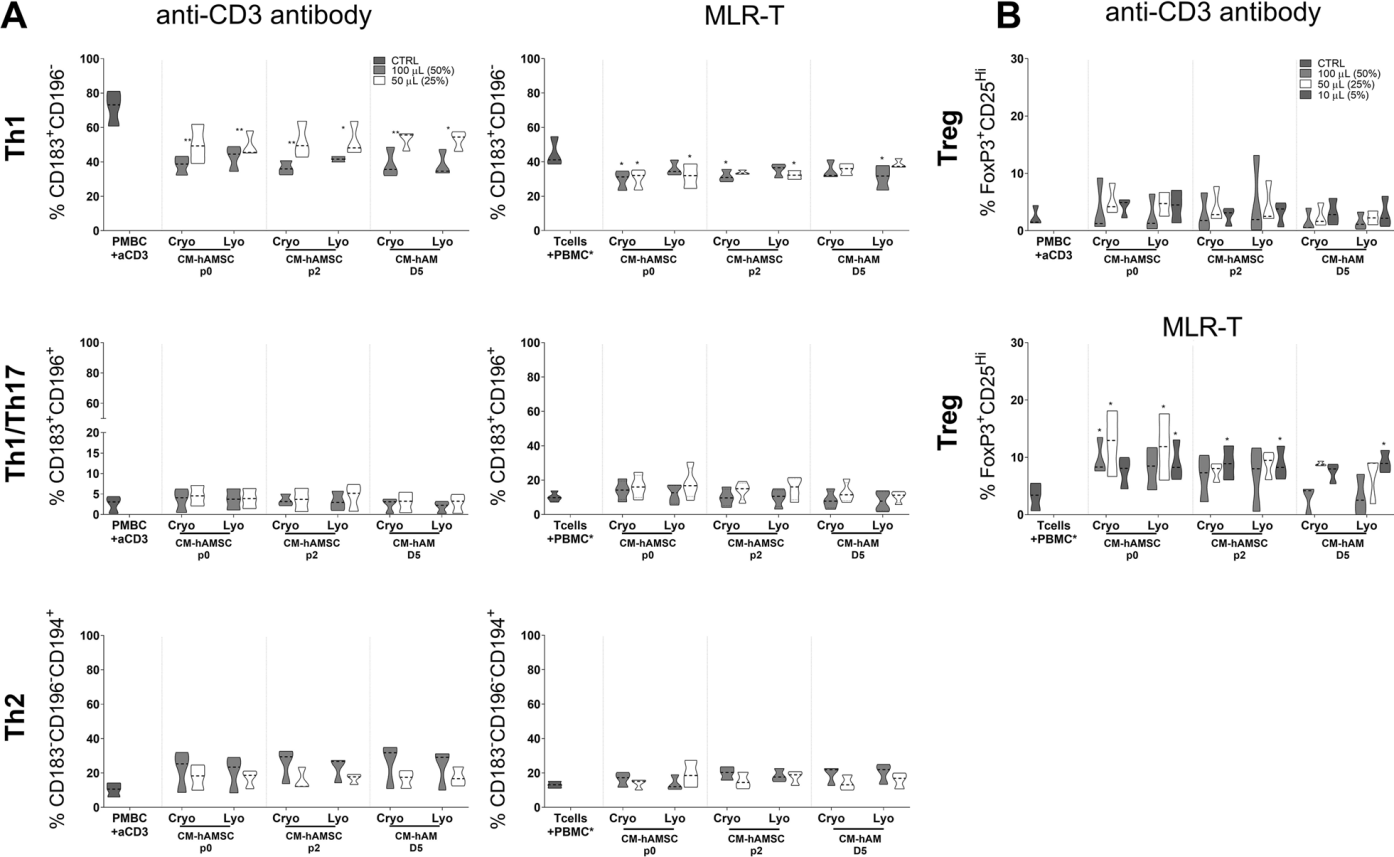
Comparison of lyophilized and cryopreserved CM on T-cell subset polarization and on Tregs. Allogeneic PBMC were stimulated with anti-CD3 mAb or in mixed T lymphocyte reaction (MLR-T) in the presence of three different concentrations of cryopreserved or lyophilized CM, 100 µL (50% of final volume), 50 µL (25%) or 10 µL (5%), from hAMSC at passage 0 (CM-hAMSC p0) or passage 2 (CM-hAMSC p2), or CM from hAM collected at 5 days after culture (CM hAM D5). **A** Different Th subsets were analyzed by flow cytometry at day 7 and expressed as a percentage of CD4+CD45RA− cells gated for Th1 (CD183+CD196−), Th1/Th17 (CD183+CD196+) and Th2 (CD183−CD196−CD194+) (in presence of two different concentrations of cryopreserved or lyophilized CM, 100 µL (50% of final volume) or 50 µL (25% of final volume), from hAMSC at passage 0 (CM-hAMSC p0) or passage 2 (CM-hAMSC p2), or CM from hAM collected at 5 days after culture (CM hAM D5). **B** Induction of Treg was evaluated by flow cytometry after six days of co-culture in allogeneic PBMC stimulated with anti-CD3 mAb (upper panel) or in mixed T lymphocyte reaction (bottom panel), in presence of 3 concentrations of cryopreserved or lyophilized CM, 100 µL (50% of final volume) or 50 µL (25% of final volume), or 10 µL (5% of final volume), and displayed as a percentage of CD45RA − FoxP3^+^CD25^hi^ cells. Results are represented as violin plots showing median (thick line), 25th and 75th quartiles (*p < 0.05, **p < 0.01, ****p < 0.0001 versus control PBMC + antiCD3 or T cells + PBMC*), *N* ≥ 3 individual experiments

In the same settings, we then analyzed whether lyophilized CMs maintained their ability to modulate T cell subsets (Fig. 5A) and to induce Treg cells [5] (Fig. 5B). In PBMC activated with anti-CD3 (Fig. 5A, left panel), all CM preparations, including lyophilized CMs, were able to significantly decrease Th1 cells (Fig. 5A, upper left panel), while all preparations were able to slightly increase the percentage of Th2 cells (Fig. 5A, lower left panel), even if this increase did not reach statistical significance. All CMs had no effect on Th1/Th17 cells (Fig. 5A, middle left panel). Similar effects were confirmed also in the MLR-T setting; however, the decrease in Th1 cells (Fig. 5A, upper right panel) was less appreciable and not significant with respect to anti-CD3 stimulation setting.

In addition, we investigated the effects of CM preparations, lyophilized and cryopreserved, on Treg cells (Fig. 5B). Interestingly, we found that the ability of CM to induce Treg varies with the T-cell stimulus applied. In the MLR-T setting (Fig. 5C, bottom panel), all CMs were able to induce Treg cells. On the other hand, CMs show a weaker, at times absent, effect in PBMC stimulated with anti-CD3 (Fig. 5B, top panel).

**Fig. 6:**
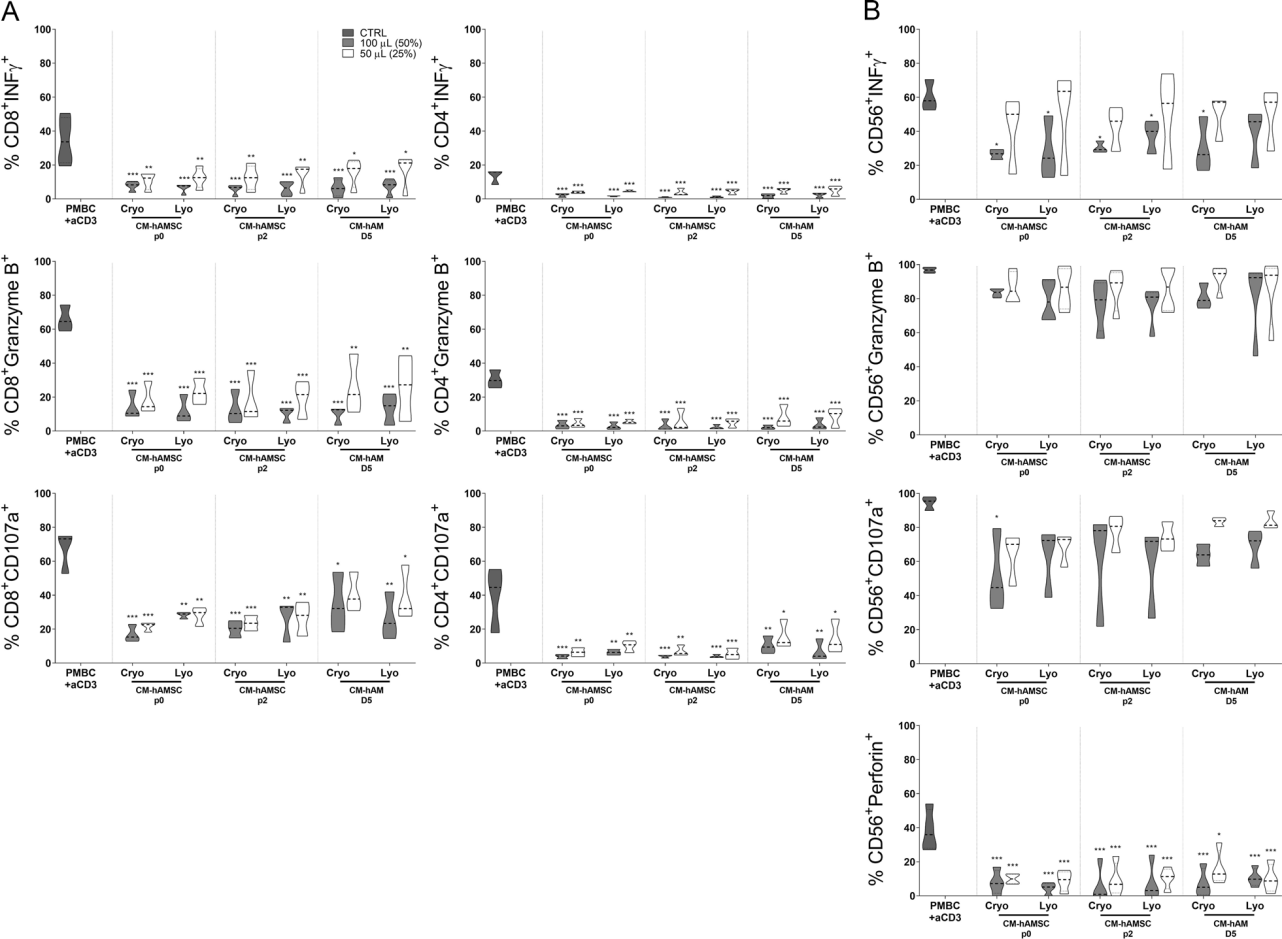
Comparison of lyophilized and cryopreserved CM on the cytotoxic activity marker expression by T lymphocytes and NK cells. **A** Allogeneic PBMC were stimulated with anti-CD3 mAb in the presence of two different concentrations of CM, 100 µL (50%) or 50 µL (25%) from hAMSC at passage 0 (CM-hAMSC p0) or passage 2 (CM-hAMSC p2), or CM from hAM collected at 5 days after culture (CM hAM D5), respectively, cryopreserved vs lyophilized. After three days of culturing, PBMC were activated with PMA + ionomycin and golgistop, brefeldin A was added 75 min later and, 4.25 h later the cells were collected and stained. The frequency of Granzyme B (GrzB +), IFN-γ and CD107a within the CD8+ and CD4+ T lymphocytes was assessed (left panel). **B** In addition to GrzB+, CD107a and IFN-γ, NK cells, defined as CD3−CD56+ cells, were analyzed for the cytotoxicity marker Perforin (Perforin+). PBMC incubated with anti-CD3 mAb, first lane of each graph, were used as controls. Results are displayed as violin plots showing median (thick line), 25th and 7th quartiles (*p < 0.05, *p < 0.01, ***p < 0.001, ****p < 0.0001 versus control (PBMC + anti-CD3), *N* ≥ 3 individual experiments

We then analyzed whether lyophilized CMs maintained the ability to reduce the cytotoxic activities of T and NK cells (Fig. 6A). We observed that all CM preparations were able to significantly decrease IFN-γ and granzyme B expression on CD8 T cells, and the effects were comparable between lyophilized and non-lyophilized CM preparations. All CM preparations similarly reduced CD4 T-cell cytotoxicity (Fig. 6A).

Next, we analyzed the ability of lyophilized CMs to reduce NK-cell cytotoxicity in terms of expression of IFN-γ, granzyme B, perforin, and CD107a (a marker of degranulation) (Fig. 6B). All CM preparations were able to strongly decrease IFN-γ and perforin expression on NK cells, while the decrease observed in granzyme B and CD107a was not significant.

**Fig. 7:**
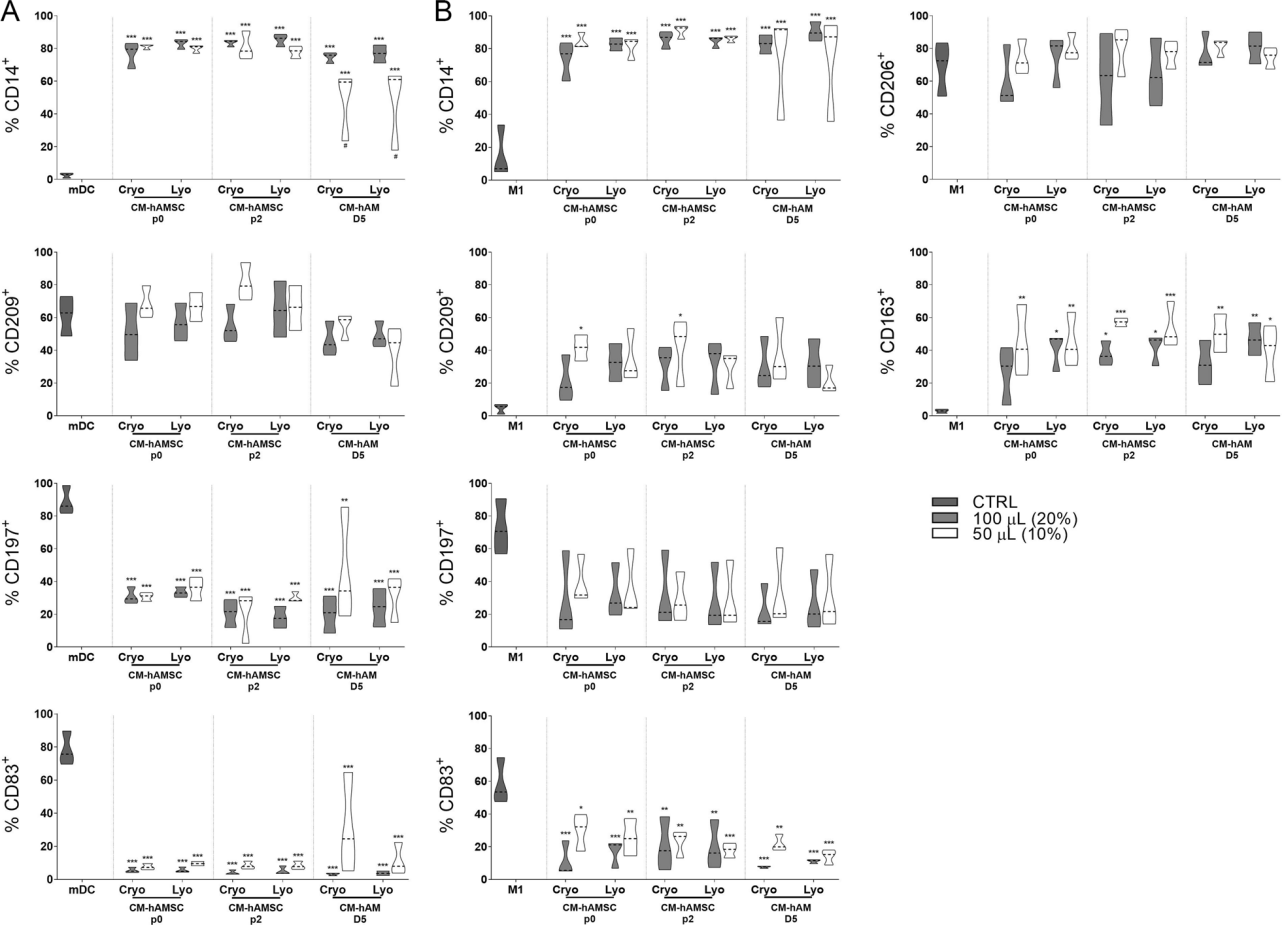
Comparison of lyophilized and cryopreserved CM on PBMC to APC differentiation. Phenotype analysis of PBMC differentiated into **A** mature dendritic cells (mDC) or **B** M1 macrophages in the presence of 100 µL or 50 µL (20% or 10% of final volume) of cryopreserved or lyophilized CM from hAMSC at passage 0 (CM-hAMSC p0) or passage 2 (CM-hAMSC p2), or CM from hAM collected at 5 days after culture (CM hAM D5). CMs were added at the start of the differentiation protocol to evaluate the ability of different products to affect monocyte differentiation toward either mDC or M1 macrophages. **A** mDC differentiation was carried out by incubating PBMC with GM-CSF + IL-4 for four days followed by two days of LPS treatment. **B** M1 macrophages were obtained by incubating PBMC with GM-CSF for four days, followed by IFN-γ + LPS for another two days. At the end of the culture period, expression of CD14, CD209, CD197 and of the co-stimulatory molecule CD83 was evaluated by flow cytometry for mDC. Furthermore, for PBMC to M1 differentiation the expression of the CD206 and CD163 markers were analyzed. Results are presented as a percentage of expression and are shown violin plots show median (thick line), 25th and 75th quartiles (*p < 0.05, **p < 0.01, ***p < 0.001, ****p < 0.0001 versus control mDC or M1), *N* ≥ 3 individual experiments

Finally, we investigated if lyophilized CMs were able to impact monocyte differentiation toward APC such as mature dendritic cells (mDC, Fig. 7A) and classically activated (M1) macrophages (Fig. 7B). All lyophilized and non-lyophilized CM preparations reduced monocyte differentiation toward mDC both by maintaining the expression of the undifferentiated monocytic marker CD14 (Fig. 7A) and by reducing the expression of markers associated with mDC differentiation, such as maturation marker CD197 (chemokine receptor CCR7) and costimulatory molecule CD83 (Fig. 7A). There were no significant effects observed on the expression of the differentiation marker CD209 (DC-SIGN) (Fig. 7A).

Similar to mDC, all CM preparations negatively impacted monocyte differentiation toward M1 macrophages. Indeed, all lyophilized and non-lyophilized CM preparations maintained a high expression of CD14 (Fig. 7B) while they significantly reduced the expression of maturation marker CD197 (chemokine receptor CCR7) and of the costimulatory molecule CD83 (Fig. 7B). All CMs not only reduced monocyte differentiation toward M1 macrophages, but they promoted monocyte differentiation toward alternatively activated (M2) macrophages. As a matter of fact, we observed a significant increase in M2 markers such as CD209 (DC-SIGN) and the gold standard marker CD163 (Fig. 7B), while no effects were observed on the expression of CD206 (Fig. 7B). In summary, all different CMs analyzed were able to interfere with the differentiation of monocytes toward M1 macrophages or mDC, while promoting monocyte polarization toward anti-inflammatory M2 macrophage subset.

## Discussion

Given the clinical relevance and the advantages of applying a cell-free therapy based on cell-secreted bioactive factors over cellular products, and the relevance of the immunomodulatory strategy in regenerative medicine [27, 28], herein we sought to identify a new cell-free product from intact hAM that has immunomodulatory properties and is able to address the following important points for clinical translation: (1) to exploit an easier production process in terms of reduced manipulation, time and costs, (2) to obtain a large volume of CM by pooling single donors that on the one hand addresses donor variability and on the other the clinical need (e.g., administration of multiple doses or treatment of large patient cohorts) and finally (3) to obtain an off-the-shelf product that is easy to store and ship.

Concerning the first point, the production of CM from intact hAM bypasses the need for cell isolation, which implies elevated production time and costs. Indeed, hAMSC isolation from hAM requires approximately 6 h and is associated with elevated reagent costs, which are further amplified when considering GMP production.

The second point, represented by the need to have a sufficient volume of CM, was addressed by pooling CM from different donors. Furthermore, the use of intact hAM has the advantage of obtaining a fivefold greater volume of CM with respect to that obtained from hAMSC isolated from the same amount of hAM. An alternative strategy to fulfill the need to obtain larger volumes of CM entails cell expansion (CM from hAMSC at passage 2).

Finally, to address the third point, we evaluated whether lyophilization could represent a valuable strategy to produce a concentrated CM guaranteeing long-term stability and easy storage. It is essential, however, that the new cell-free product from intact hAM retains the unique properties reported for CM from hAMSC. Despite the large number of studies focused on exploring the in vitro and in vivo properties of CM derived from the culture of cells isolated from hAM [4], very few studies have investigated the properties of CM from the intact hAM, and specifically, they show its ability to inhibit tumor cell proliferation [29] and to promote healing in corneal diseases [30–32]. To our knowledge, no study has ever investigated in depth the immunomodulatory properties of CM from intact hAM and compared them with CM from hAMSC.

In this study, with the purpose to compare the different CM, we evaluated, as proposed by Galipeau's group [33], the CM’s in vitro activities on different types of immune cells belonging to innate and adaptive immunity, therefore providing a comprehensive and detailed characterization of their immunomodulatory properties.

Here for the first time, we demonstrate that CM from hAM, similar to CM from hAMSC, inhibits T cell proliferation, modulates the polarization of T lymphocytes, induces regulatory T cells, inhibits the expression of different cytotoxicity markers on CD4, CD8 and NK cells, and inhibits the differentiation of monocytes toward mature antigen-presenting cells (i.e., dendritic cells and M1 macrophages), concomitantly promoting M2 polarization.

It should be underlined that the inhibitory effects observed for the CMs tested herein do not depend on cytotoxic factors released from hAM or hAMSC, nor on nutrient depletion due to in vitro culture. Indeed, different CMs did not affect cell viability, but impacted only cell activation or differentiation processes. Moreover, CMs produced from the in vitro culture of other cell types, such as MSC from bone marrow and dermal fibroblasts, have also been reported to have no effect on PBMC proliferation ([25] and unpublished data). On the other hand, amniotic CMs’ effects are conserved even after they are supplemented with FBS and essential and nonessential amino acids [34].

Furthermore, here we report that the effect of CM from expanded cells is comparable to that from non-expanded cells. Concerning the reduction in time and costs, the use of CM from hAM bypasses the need for cell isolation thus reducing manipulation and possible cell alterations, while maintaining a comparable efficacy to the CM obtained from hAMSC. Furthermore, CM collected from hAM left only 2 days in culture medium presents an immunomodulatory effect, differently from what was previously reported for CM-hAMSC, where the immunomodulatory effect was observed only from CM derived from hAMSC maintained in culture for at least 4 days [34]. This could in part be explained by the fact that hAM is not subject to enzymatic digestion (as is the case when hAMSC are used), and therefore, the cells could maintain higher viability and functionality. In addition, intact hAM also contains epithelial cells that have been reported to also have immunomodulatory potential [8, 35].

Moreover, for the first time we demonstrate that CM immunomodulatory capabilities are maintained regardless of whether CM derives from single donor preparations or from pooled ones confirming that pooling CM can be a strategy to address large volumes for clinical translation. As a matter of fact, cell expansion, although allowing for the production of larger volumes of CM, does not address the need for simplifying the production process and reducing costs.

We should underline the fact that CMs and pooled CMs are biological material and all possible measures should be applied to verify the identity, potency, purity and safety of all batches as recommended by global regulatory agencies and European Pharmacopeia. Specifically, for pooled “material,” limiting the size of donors has been recommended in order to control the risk to transmitted pathogens [36] and the assurance of the traceability from donor to recipient (European Pharmacopeia (chapter 5.2.12)).

Finally, we demonstrate that lyophilization does not alter the immunomodulatory properties of CM. Other groups have reported that lyophilization does not alter functional properties of CM [37], showing that lyophilized CM from corneal MSC was just as efficient as fresh CM in promoting wound healing and in decreasing the mRNA expression of inflammatory genes (ICAM 1, TLR3, IL-6, IL-8, and TNF-α) in stimulated macrophages.

Even though lyophilization represents an interesting approach toward an off-the-shelf product, quality controls must be carefully considered since inadequate lyophilization can lead to a serious alteration of the immunomodulatory factors; thus, an optimal lyophilization protocol must be defined to translate amniotic CMs to the clinic.

## Conclusions

The results presented herein open the possibility to highly improve the scalability of CM during the GMP production process while reducing the costs and time related to the process of isolation of MSC and to the possible expansion of the cells. Moreover, the possibility of having a lyophilized product that maintains its original properties, and here we focused on immunomodulation, would allow for a ready-to-use product with easier handling, shipping and storage and will also allow for customized reconstitution according to the most suitable formula required by the clinical application.

To date, the proposed potency assays are limited to a partial or indirect evaluation of immunomodulation, mainly focused on PBMC proliferation or on cytokine production [23].

The panel of in vitro tests used in this study to evaluate the immunomodulatory properties of amniotic products could be useful to readdress/redesign a new multi-assay system to characterize the immune potency of MSC and their products.

## Data Availability

The datasets generated and/or analyzed during the current study are available from the corresponding author on reasonable request.

## Abbreviations

hAM: Amniotic membrane of human term placenta
MSC: Mesenchymal stromal cells
hAMSC: Mesenchymal stromal cells from the amniotic membrane of human term placenta
CM: Conditioned medium
CM-hAMSC: Conditioned medium from mesenchymal stromal cells from the amniotic membrane
PBMC: Peripheral blood mononuclear cells
PBS: Phosphate-buffered saline
P: Penicillin
S: Streptomycin
FBS: Fetal bovine serum
CuAAC: Cu-catalyzed alkyne-azide cycloaddition
EdU: Ethynyl-labeled deoxyuridine
CFSE: Carboxyfluorescein succinimidyl ester
PF: Proliferative fraction
MLR: Mixed lymphocyte reaction
Th: T-helper cells
Treg: T regulatory cells
PMA: Phorbol myristate acetate
IFN-γ: Interferon gamma
Mo: Monocytes
APC: Antigen-presenting cells
M1: Macrophage type 1
M2: Macrophage type 2
DC: Dendritic cells
mDC: Mature dendritic cells
NK: Natural killer cells
LPS: Lipopolysaccharide
GM-CSF: Granulocyte macrophage colony stimulating
ANOVA: Analysis of variance
Lyo: Lyophilized
